# A Compend of Pharmacy

**Published:** 1886-09

**Authors:** 


A COMPEND OF PHARMACY. By F. E. Stewart, M. D., Ph. G. Quiz-master in Chemistry and Theoretical Pharmacy in the Philadelphia College of Pharmacy; Lecturer on and Demonstrator of Pharmacy in the Medico-Chirurgical College of Philadelphia; Demonstrator of Pharmacy in the Woman's Medical College of Pennsylvania; member of the American Pharmaceutical Association, etc. Based upon Prof. Joseph P. Remington's "Text-Book of Pharmacy." Philadelphia: P. Blakiston Son & Co. 1886.

This Compend of Pharmacy is based on Prof. Joseph P. Remington's "Text-Book of Pharmacy," which is now the acknowledged standard authority. The author and publishers have given much time towards making it as complete and concise as possible, and in arranging the types and paragraphs so that it may be easily and quickly referred to. It will be found a useful guide to the worker in pharmacy.

